# Evaluation of the Endorsement of the STrengthening the REporting of Genetic Association Studies (STREGA) Statement on the Reporting Quality of Published Genetic Association Studies

**DOI:** 10.2188/jea.JE20150173

**Published:** 2016-08-05

**Authors:** Darko Nedovic, Nikola Panic, Roberta Pastorino, Walter Ricciardi, Stefania Boccia

**Affiliations:** 1 Section of Hygiene, Institute of Public Health, Università Cattolica del Sacro Cuore, Rome, Italy; 2 Faculty of Medicine, University of Nis, Nis, Serbia; 3 University Clinical-Hospital Center “Dr Dragisa Misovic-Dedinje”, Belgrade, Serbia

**Keywords:** genetic association studies, STREGA, quality, genetics and heredity

## Abstract

The STrengthening the REporting of Genetic Association studies (STREGA) statement was based on the STrengthening the REporting of OBservational studies in Epidemiology (STROBE) statement, and it was published in 2009 in order to improve the reporting of genetic association (GA) studies. Our aim was to evaluate the impact of STREGA endorsement on the quality of reporting of GA studies published in journals in the field of genetics and heredity (GH). Quality of reporting was evaluated by assessing the adherence of papers to the STREGA checklist. After identifying the GH journals that endorsed STREGA in their instructions for authors, we randomly appraised papers published in 2013 from journals endorsing STREGA that published GA studies (Group A); in GH journals that never endorsed STREGA (Group B); in GH journals endorsing STREGA, but in the year preceding its endorsement (Group C); and in the same time period as Group C from GH journals that never endorsed STREGA (Group D). The STREGA statement was referenced in 29 (18.1%) of 160 GH journals, of which 18 (62.1%) journals published GA studies. Among the 18 journals endorsing STREGA, we found a significant increase in the overall adherence to the STREGA checklist over time (A vs C; *P* < 0.0001). Adherence to the STREGA checklist was significantly higher in journals endorsing STREGA compared to those that did not endorse the statement (A vs B; *P* = 0.04). No significant improvement was detected in the adherence to STREGA items in journals not endorsing STREGA over time (B vs D; *P* > 0.05). The endorsement of STREGA resulted in an increase in quality of reporting of GA studies over time, while no similar improvement was reported for journals that never endorsed STREGA.

## INTRODUCTION

The number of publications reporting on the association between genetic polymorphisms and diseases has increased tremendously, reaching an annual number of more than 10 500 in 2013.^1^ Inadequate reporting of results, however, hampers the assessment of a study’s strengths and weaknesses and hence the integration of the evidence.^2^

The STrengthening the REporting of Genetic Association studies (STREGA)^3^ initiative, based on the Strengthening the Reporting of Observational Studies in Epidemiology (STROBE) Statement,^4^ was developed in order to guide the drafting of genetic association studies and increase the quality of their reporting. The STREGA checklist includes a clear description of the key elements of the study design, eligibility criteria, relevant dates, and characteristics of the study population. However, despite the many similarities between genetic association studies and conventional observational epidemiological studies, the former present several specific challenges, including population stratification, genotyping, Hardy-Weinberg equilibrium, and the rationale of choice of genes and variants and of genetic models used. Additionally, even though STREGA recommendations do not prescribe how a genetic association study should be designed, it is expected that, in the long term, these recommendations will positively influence the conception and design of genetic association studies.

Since the release of STREGA, however, no attempts have been made to evaluate whether STREGA endorsement has affected the actual quality of reporting. We conducted the present study to evaluate the impact of STREGA endorsement on the quality of reporting of genetic association studies published in scientific journals in the field of genetics and heredity.

We evaluated: i) whether STREGA endorsement led to a change in the quality of reporting of genetic association studies over time; ii) whether the quality of reporting of genetic association studies differed between journals that endorsed STREGA and those that did not; and iii) whether changes in drafting genetic association studies over time was likely attributable to STREGA endorsement or to a general improvement in drafting.

## METHODS

### Assessing endorsement of STREGA in genetics and heredity journals

The list of medical journals in the field of genetics and heredity (GH) was acquired from Thomson Reuters ISI Web of Knowledge using the proper code field by accessing http://science.thomsonreuters.com/cgi-bin/jrnlst/jlresults.cgi?PC=D&SC=KM in January 2013. Two assessors (DN and NP) independently examined the websites of the 160 journals retrieved, searching for any mention of STREGA. The search was performed between November 1 and December 1, 2013. For each journal, we retrieved the impact factor value according to the Journal Citation Reports 2012. Editors of journals who endorsed STREGA were then contacted to determine the exact year of STREGA endorsement in the instructions for authors.

### Identification of genetic association studies published in GH journals

Among 160 GH journals, 29 (18.1%) endorsed STREGA in their instructions for authors. However, we excluded 11 of these because we could not identify any genetic association studies published in 2013 on MEDLINE, ultimately including 18 GH journals.

In order to select the papers to appraise, we used the following search terms in MEDLINE: ([journal name] AND [“genetic association”]). The journal names were the 18 included journals, and the time frame varied according to the comparisons reported below. After listing all papers identified in each journal, we used a random numbers table to select 1 paper published in 2013 from each of the 18 journals endorsing STREGA (Group A) and an identical number of papers published in 2013 among 18 GH journals that never endorsed STREGA (Group B). Journals in Group B were matched to Group A according to the closest 2012 impact factor value.

**Figure 1.**
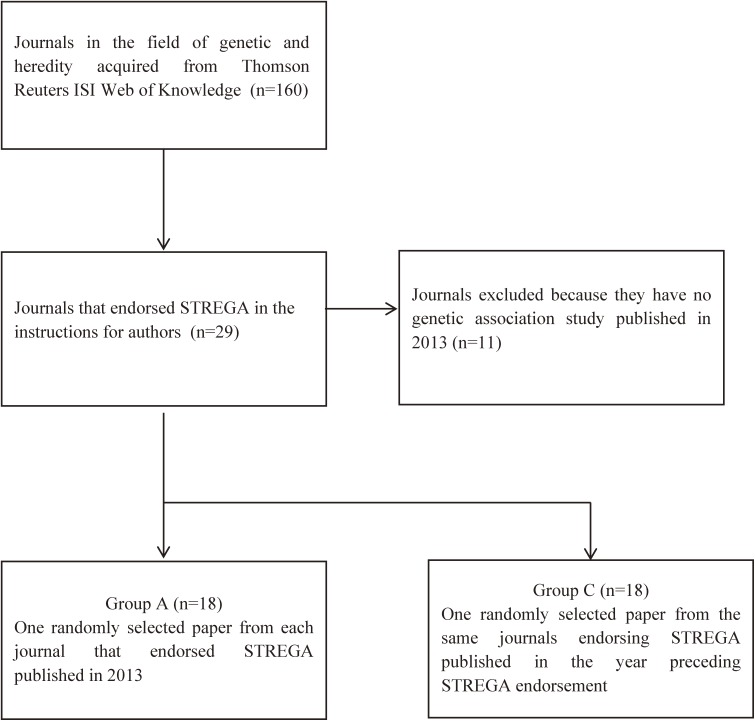
Flowchart of database search of genetic and heredity journals for the identification of papers included in Group A and Group C.

**Figure 2.**
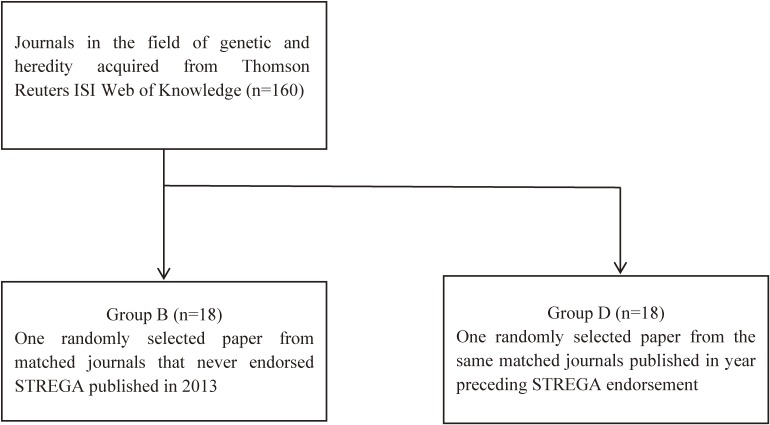
Flowchart of database search of genetic and heredity journals for the identification of papers included in Group B and Group D.

In order to assess whether STREGA endorsement led to an improvement in drafting over time, we additionally appraised 18 papers randomly selected from the same journals endorsing STREGA that were published in the year immediately preceding STREGA endorsement (Group C), as well as 18 randomly selected papers published in the same time period from their matched GH journals (one paper per journal) that never endorsed STREGA (Group D). Figure 1 and Figure 2 depict the flow charts of the search strategies used to identify the four groups.

We assessed the quality of reporting by evaluating the adherence of the papers to the STREGA checklist. The STREGA checklist contains 22 items, which are grouped in order to address the quality of reporting in “Title and Abstract”, “Methods”, “Results”, “Discussion”, and funding. Scoring the papers with the STREGA checklist was performed by two researchers separately (DN and NP); in case of disagreement, an independent third researcher (SB) was consulted. Our a priori research aims were to assess the following: i) whether the adoption of STREGA improved the quality of reporting of genetic association studies over time (comparison of groups A vs C); ii) whether quality of reporting of published genetic association studies differed among journals endorsing STREGA compared to those not endorsing STREGA during the same time period (comparison of groups A vs B); and iii) whether any difference in reporting over time was likely attributable to a general improvement in drafting or was indeed related to STREGA adoption (comparison of groups B vs D).

### Statistical analysis

We defined adequate reporting as adhering to at least 80% of the given recommendations per item. If an item was given by only one recommendation we defined adequate reporting in cases where the item itself was addressed satisfactory. Comparison of the data between groups was evaluated using the chi-square or Fisher’s test, as appropriate. Statistical analysis was undertaken using Stata software version 12 (StataCorp, CollegeStation, TX, USA).

## RESULTS

### Assessing endorsement

Out of 160 GH journals identified, 29 (18.1%) endorsed STREGA in their instructions for authors, of which 18 (62.1%) published genetic association studies. Journal names were “American Journal of Human Genetics”, “PLoS Genetics”, “Genetics in Medicine”, “BMC Genomics”, “Clinical Genetics”, “Genome Medicine”, “Pharmacogenetics and Genomics”, “Genetics Selection Evolution”, “BMC Medical Genomics”, “BMC Evolutionary Biology”, “BMC Genetics”, “BMC Medical Genetics”, “Journal of Human Genetics”, “Cancer Genetics”, “G3-Genes Genomes Genetic”, “Genetic Testing and Molecular Biomarkers”, “Ophthalmic Genetics”, and “Psychiatric Genetics”. Among these journals, 6 (33.4%) endorsed the STREGA statement in 2009, 10 (55.5%) endorsed the statement in 2010, and 2 (11.1%) endorsed the statement in 2012. There was no difference in the mean impact factor value among the 29 journals endorsing STREGA and those that did not (*P* = 0.82; data not shown).

### Critical appraisal of genetic association studies published in genetic and heredity journals

We evaluated four groups consisting of 18 studies each. Of the studies included, 30.6% were genome-wide association studies, while 69.4% were genetic association studies. The Table reports the proportion of studies with adequate reporting of the 22 items of the STREGA statement in the articles analyzed and the overall adherence for each group.

**Table.** Proportion of adequate reporting of the 22 items of the STREGA statement in the articles appraised within the four groups and comparison between the groups (A vs B, A vs C, B vs D)

STREGA	Group A	Group B	Group C	Group D
Item				
1	100.0	83.3	94.4	61.1
2	100.0	100.0	100.0	100.0
3	100.0	100.0	94.4	100.0
4	100.0	100.0	100.0	100.0
5	83.3	77.8	77.8	77.8
6	61.1	66.7	38.9	72.2
7	38.9	16.7	5.6*	0.0
8	22.2	22.2	0.0*	11.1
9	44.4	22.2	11.1	16.7
10	61.1	11.1*	11.1*	100.0*
11	83.3	83.3	50.0*	0.0*
12	11.1	0.0	0.0	0.0
13	11.1	0.0	0.0	0.0
14	5.6	0.0	0.0	0.0
15	100.0	100.0	83.3	100.0
16	5.6	0.0	0.0	0.0
17	16.7	0.0	0.0	0.0
18	100.0	100.0	100.0	100.0
19	66.7	55.6	38.9	50.0
20	100.0	100.0	100.0	100.0
21	83.3	100.0	83.3	88.9
22	100.0	100.0	100.0	100.0
Total (95% CI)	63.0 (59.0–68.0)	56.0 (51.0–61.0)*	49.0 (45.0–54.0)*	54.0 (49.0–58.0)

CI, confidence interval; STREGA, The STrengthening the REporting of Genetic Association studies statement.

*P < 0.05; *P*-value calculated for each of these comparisons A vs B, A vs C, and B vs D.

In order to assess whether STREGA endorsement led to an increase in the quality of reporting of genetic association studies published over time, we compared group A to group C and found a significant increase in the overall adherence to STREGA (63.0% versus 49.0%; *P* < 0.0001) (Table).

For specific items, we found a significantly higher rate of adherence between group A and group C for item 7 of STREGA, “Variables” (38.9% versus 5.6%; *P* = 0.016); item 8, “Data sources measurement” (22.2% versus 0.0%; *P* = 0.034); item 10, “Study size” (61.1% versus 11.1%; *P* = 0.002); and item 11, “Quantitative variables” (83.3% versus 50.0%; *P* = 0.034) (Table).

In order to assess whether quality of reporting and methodological quality of published genetic association studies differed among journals endorsing STREGA compared to those not endorsing the statement, we compared groups A and B. We observed significantly higher overall adherence to STREGA in Group A (63.0% versus 56.0%; *P* = 0.04) (Table). In particular, STREGA item 10, “Study size” (61.1% versus 11.1%; *P* = 0.002), was addressed correctly significantly more often in Group A (Table).

Lastly, in order to assess whether the observed improvement in quality of reporting in journals endorsing STREGA is attributable to STREGA adoption, we compared groups B and D. As we observed no significant improvement in adherence to STREGA items in journals not endorsing STREGA over time (56.0% versus 54.0%; *P* = 0.40) (Table), the observed improvement in quality of reporting in journals endorsing STREGA can be attributed to STREGA endorsement.

## DISCUSSION

Although a relatively small proportion of the scientific journals in the field of GH endorsed STREGA in their instructions for authors, we found that the quality of reporting of genetic association studies in these journals significantly increased after STREGA endorsement. Such improvement in paper drafting was not observed in GH journals not endorsing STREGA, suggesting that the checklist itself was likely to be the cause of the observed improvement in reporting. Lastly, the quality of reporting was higher in journals endorsing STREGA compared to those not endorsing the statement during the same time period.

Complete and transparent reporting of research studies is an important foundation of knowledge translation.^5^ Lack of transparency and incomplete reporting have raised concerns in a range of health research fields,^6^^,^^7^ and poor reporting has been associated with biased estimates of effects in clinical intervention studies.^8^ Even an excellently designed and conducted study is of limited value if it is inadequately reported. Aspects of good publication practice, such as the use of reporting guidelines, have been promoted for years now. Reporting guidelines and online supplemental material may help enhance the transparency of genome epidemiology association studies.^9^ Nevertheless, the uptake and implementation by journals publishing in the field of GH is generally insufficient. Our study found that a small portion of GH journals endorsed STREGA in their instructions for authors.

Little is known about the most effective ways to apply reporting guidelines in practice, so editors and authors have been encouraged to collect, analyse, and report their experiences in using such guidelines.^10^ The impact of guidelines on quality of published papers in medical journals has so far been contradictory. It has been reported that endorsement of the Consolidated Standards for Reporting Trials (CONSORT)^11^ has led to improved quality in the reporting of randomized clinical trials.^12^^–^^14^ On the other hand, after endorsement of the STROBE statement,^4^ the quality of reporting of published observational studies was still reported as unsatisfactory.^15^^,^^16^ We recently reported that endorsement of the Preferred Reporting Items for Systematic Reviews and Meta-Analysis (PRISMA) statement^17^ led to an increase in both methodological quality and quality of reporting of systematic reviews and meta-analyses.^18^ Prior to STREGA,^3^ no guideline addressed the issue of drafting reports of genetic association studies. Any previous guidance in this field was focused on genetic association studies of specific diseases^19^^–^^21^ or the design and conduct of genetic association studies,^22^^–^^24^ rather than on the quality of reporting. It was expected that STREGA would bring uniform standards in drafting and lead to an increase in the quality of reporting. No study so far, however, has attempted to assess the impact of STREGA endorsement on quality of reporting of genetic association studies.

Results of our study show that quality of reporting, measured as overall adherence to STREGA checklist items, improved in journals endorsing STREGA. Furthermore, improvement is likely attributable to STREGA itself, as we did not observe improvement in the overall reporting quality of published genetic association studies in journals not endorsing STREGA.

Finally, where we observed an increase in the adherence to specific items of STREGA, some were items having a major impact on the quality of reporting, such as variables (eg, using widely-used nomenclature systems to define genetic exposures), data source measurements (eg, genotyping methods and platforms, error rates, and call rates), and study size. However adherence to other crucial elements, such as adequate description of statistical methods, study participants, descriptive data on participants, presentation of main results, and other analyses, was very low, and no improvement was noted. Therefore, we suggest that it is not sufficient for journals to simply recommend the use of STREGA to authors in the authors’ instructions; instead, journals should require submission of the STREGA checklist together with the manuscript. In this way, better reporting of items on the STREGA checklist that now appear to be insufficient could be achieved.

There are some limitations to our study. First, we know that not all journals in the field of GH are indexed in Journal Citation Reports 2012. Therefore, our evaluation is not fully representative of all existing journals in this field. Second, we randomly selected one paper per journal, which could limit our findings due to the small number of assessed studies. Last, we could not assess whether the STREGA endorsement impacted the quality of the conduct of genetic association studies. Even though improving the quality of study conduct was not intended, authors might use STREGA to assist in study design and data analyses in genetic association studies. However, we were unable to address this issue, as currently there is no validated scale to appraise genetic association studies.

In conclusion, we report that scientific journals in the field of genetics and heredity that endorsed STREGA had an improvement in the quality of reporting of genetic association studies over time. STREGA should be endorsed in the instruction for authors of a larger proportion of scientific journals publishing genetic association studies.
